# Detection and prognostic relevance of PLA2R epitopes in idiopathic membranous nephropathy: a simultaneous quantitative multiplex suspension array detection method

**DOI:** 10.1093/ckj/sfaf010

**Published:** 2025-01-13

**Authors:** Juan Wu, Qingjuan Zhang, Yuanyuan Du, Tianyu Zheng, Juan Jin, Shangbin Kao, Xiumei Zhou, Yuan Qin, Xueqin Zhao, Qiang He, Fuzhou Yang, Biao Huang

**Affiliations:** College of Life Sciences and Medicine, Zhejiang Sci-Tech University, Hangzhou, Zhejiang, China; Department of Nephrology, The Affiliated Jiangning Hospital with Nanjing Medicine University, Nanjing, Jiangsu, China; Department of Nephrology (Key Laboratory of Kidney Disease Prevention and Control Technology), Hangzhou TCM Hospital Affiliated to Zhejiang Chinese Medical University, Hangzhou, China; College of Life Sciences and Medicine, Zhejiang Sci-Tech University, Hangzhou, Zhejiang, China; Department of Nephrology, the First Affiliated Hospital of Zhejiang Chinese Medical University (Zhejiang Provincial Hospital of Traditional Chinese Medicine), Hangzhou, Zhejiang, China; Provincial Key Laboratory for Research and Translation on the Syndrome of Kidney Deficiency Accompanied by Blood Stasis and Turbidity, Hangzhou, China; College of Life Sciences and Medicine, Zhejiang Sci-Tech University, Hangzhou, Zhejiang, China; College of Life Sciences and Medicine, Zhejiang Sci-Tech University, Hangzhou, Zhejiang, China; College of Life Sciences and Medicine, Zhejiang Sci-Tech University, Hangzhou, Zhejiang, China; College of Life Sciences and Medicine, Zhejiang Sci-Tech University, Hangzhou, Zhejiang, China; Department of Nephrology, the First Affiliated Hospital of Zhejiang Chinese Medical University (Zhejiang Provincial Hospital of Traditional Chinese Medicine), Hangzhou, Zhejiang, China; Provincial Key Laboratory for Research and Translation on the Syndrome of Kidney Deficiency Accompanied by Blood Stasis and Turbidity, Hangzhou, China; Department of Nuclear Medicine, Ya’an People's Hospital, Ya’an, Sichuan, China; College of Life Sciences and Medicine, Zhejiang Sci-Tech University, Hangzhou, Zhejiang, China; Provincial Key Laboratory for Research and Translation on the Syndrome of Kidney Deficiency Accompanied by Blood Stasis and Turbidity, Hangzhou, China

**Keywords:** epitope spreading, IgG4 antibody, IMN, multiplex suspension assay, PLA2R

## Abstract

**Objective:**

This study aimed to develop a multiplex suspension assay for the simultaneous quantitative detection of anti-cysteine-rich domain (anti-CysR)/C-type lectin domain 1 (CTLD1)/C-type lectin domain 6–8 (CTLD678)–immunoglobulin G4 (IgG4) antibodies of M-type phospholipase A2 receptor (PLA2R) in the serum samples of patients to evaluate the clinical application value of PLA2R epitope spreading in disease prognosis.

**Methods:**

CysR, CTLD1 and CTLD678 antigen domains were coupled to three types of microspheres. The optimal dilution ratio of biotinylated anti-human IgG4 was identified, and a multiplex suspension assay was evaluated in terms of linearity, sensitivity, precision, specificity and recovery rate. Lastly, the relationship between epitope spreading and the severity of idiopathic membranous nephropathy was evaluated.

**Results:**

The content of all three epitopes could be detected simultaneously within 2 h. The intra-assay precision ranged from 4.74% to 9.48%, and the inter-assay precision was between 5.13% and 13.92%. Specific experiments showed that the human IgA antibody did not cause a cross-reaction. The recovery rates ranged between 90% and 100%. The cut-off values of epitopes CysR, CTLD1 and CTLD678 between healthy individuals and patients were 6.30, 12.38 and 10.06 Ru/mL, respectively. Among them, the positive rates of epitopes CysR, CTLD1 and CTLD678 were 100%, 34% and 75%, respectively. In addition, Group 3 (CTLD1 response) accounted for 73% of the 12 patients who were not in remission. Meanwhile, when the concentration of CTLD1 exceeds 42.76 Ru/mL, the patient's prognosis may be poorer.

**Conclusion:**

A multiplex suspension assay was developed for the simultaneous quantitative detection of anti-CysR/CTLD1/CTLD678–IgG4 antibodies. The epitope migration sequence of PLA2R molecules during disease progression may not follow a simple linear rule. Among them, the epitope CTLD1 is likely to exert the most significant influence on patient remission.

KEY LEARNING POINTS **What was known:**

- The detection method can keep the serum detection environment consistent, allowing more accuracy.

**This study adds:**

- The analysis shows that the migration sequence of PLA2R epitopes may not follow the linear rule of previous studies from CysR to CTLD1 and finally to CTLD678, but there is a hopping phenomenon.

**Potential impact:**

- The results showed that the concentration of epitope CTLD1 may be correlated with the disease severity of idiopathic membranous nephropathy.

## INTRODUCTION

Membranous nephropathy (MN) is an autoimmune glomerular disease that can occur in all ages [1–3]. Meanwhile, idiopathic MN (IMN) is a glomerular disease caused by a kidney-specific autoimmune reaction. Patients with IMN are often diagnosed with severe proteinuria (content >3.5 g/24 h), hypoalbuminemia (content <30 g/L), hyperlipidemia and other corresponding clinical manifestations. The main mechanism involved in this disease is the accumulation of immune deposits outside the glomerular basement membrane, leading to the thickening of the basement membrane. The main components of immune deposits are immunoglobulin G (IgG) and complement [4–6].

M-type phospholipase A2 receptor (PLA2R) was discovered in 2009 as the main target of MN [7]. PLA2R is considered the main antigen involved in the pathogenesis of IMN. It is a 185-kDa membrane receptor glycoprotein with 10 distinct 7–17 kDa globular domains in its extracellular region, namely, cysteine-rich domain (CysR), fibronectin type II domain and eight distinct C-type lectin domains (CTLD1–8). It also contains a transmembrane domain and an intracellular C-terminal tail. Antibodies against PLA2R mainly belong to the IgG4 subclass [8–10]. The immunodominant epitope of PLA2R is in the N-terminal CysR domain [9, 11, 12]. The other two antigenic domains are CTLD1 and CTLD7 [10, 13]. In 2020, Reinhard *et al*. [14] identified the fourth epitope targeted by the PLA2R antibody and constructed and expressed the PLA2R1 Deletion Constructs by characterizing the domain recognized by the PLA2R antibody. The CTLD8 domain at the C-terminal of PLA2R was identified as the fourth immunoreactivity epitope of PLA2R.

The phenomenon “epitope spreading” in patients with IMN has been proposed. Epitope response in PLA2R may be related to the prognoses of patients and can indicate disease activity early and accurately. IMN identified only in the CysR domain has a high possibility of spontaneous remission. Conversely, patients whose PLA2R autoantibodies have been identified in the CTLD1, CTLD7 or CTLD8 domains—that is, patients with epitope spreading, have worse prognoses, more severe albuminuria and are more likely to develop end-stage renal disease than those without [3, 15]. Epitope spreading can be an independent risk factor for non-remission in patients with IMN, however this idea remains controversial. Some studies have shown that, given that patients are immunoreactive to multiple domain epitopes during clinical diagnosis [9], therefore, epitope spreading does not play a major role in the treatment and prognosis of the disease.

A previously used method for studying the different reactivity epitopes of the PLA2R antigen is enzyme-linked immune sorbent assay (ELISA), but it is mainly a semiquantitative analysis method. Although time-resolved fluorescence immunoassay (TRFIA) is a quantitative analysis method, it needs to detect different epitopes separately [8]. Given that CTLD7 or CTLD8 expression is low, CTLD678 was used as the recombinant antigen in this study. We focused on detecting IgG4 levels in the anti-CysR/CTLD1/CTLD678 domain because IgG4 plays a more prominent role than IgG in the pathogenesis of IMN [9, 15, 16].

A highly sensitive method for quantitatively detecting anti-CysR/CTLD1/CTLD678–IgG4 under consistent reaction conditions was established, which is of great importance to the study of PLA2R epitope spreading.

## MATERIALS AND METHODS

### Materials

The recombinant antigens of CysR, CTLD1, CTLD678 and mouse anti-human IgG4 antibody were purchased from Zhejiang Bosh Biological Co., Ltd. Biotin, N-hydroxy succinimide (NHS) and 1-(3-dimethylaminopropyl)-3-ethylcarbodiimide hydrochloride (EDC) were purchased from Zhejiang Boshi Biological Co., Ltd. Streptavidin–phycoerythrin (SAPE) and magnetic carboxyl fluorescent coding microspheres (numbers 33, 52 and 7) were purchased from Sigma-Aldrich. The sheath liquid, Luminex 200, Bio-Plex flat bottom template, magnetic frame and magnetic board washing machine were purchased from Bio-Rad. Other chemicals and reagents were analytically pure.

### Buffer composition

The buffer solutions were as follows: phosphate-buffered saline (PBS) (20 mmol/L, pH 7.4); coupling buffer (50 mmol/L MES, pH 5.0); assay buffer (PBS, 1% bovine albumin, pH 7.4); microsphere wash buffer [PBS, 0.1% BSA, 0.02% (v/v) Tween-20, 0.05% (v/v) Procling300, pH 7.4]; and microsphere preservation buffer [PBS, 1% BSA, 0.05% (v/v) Procling300, pH 7.4].

### Plasma samples

We collected serum samples from 20 healthy subjects and 47 patients with MN at the Hangzhou Hospital of Traditional Chinese Medicine. Each patient underwent a kidney biopsy and pathological examination during blood collection. Serum samples were collected after centrifugation at 3000 × *g* for 5 min and stored at –20°C. The serum specimen did not experience hemolysis or lipemia during the preparation process. These samples were clearly diagnosed as MN through histological or cytological examination. Diagnosis was conducted using renal biopsy techniques, including light microscopy, immunohistochemistry and immunofluorescence. Secondary causes of MN, such as hepatitis B virus, malignancies, systemic lupus erythematosus, infections and drug-induced factors, were systematically excluded. Once MN was confirmed, the laboratory performed tests for MN-specific target antigens, and no positive results were found for THSD7A, NELL-1, SEMA3B, EXT1/2 or NEP/MME. These findings established that the samples were representative of IMN. All samples were followed up consecutively to exclude the non-PLA2R-related MN group as an early stage of PLA2R-positive MN. Detailed information on the demographic characteristics of all the patients is provided in Table 1.

**Table 1:** Demographics results at the time of kidney biopsy.

Variables	Overall (*n* = 32)
Exclusion criteria	
Lupus nephritis, vasculitis infections, drug-induced	Excluded
Baseline data	
Age, years	52 ± 14
10–<30	20 ± 3
30–<50	43 (40–48)
50–<60	54 (52–56)
60–<80	63 (61–70)
Male, *n* (%)	19 (59.3)
Comorbid condition, *n* (%)	
Diabetes mellitus	8 (25.0)
Hypertension	15 (46.8)
Laboratory variables	
Serum creatinine, µmol/L	80.6 (56.3–90.2)
eGFR, mL/min/1.73 m^2^	86.9 ± 26.6
Serum albumin, g/L	24.5 (22.8–27.2)
24-h urine protein, g/day	5.2 (2.7–6.6)
Serum IgG, g/L	7.3 (5.2–8.3)
Serum IgA, g/L	2.2 (1.2–3.2)
Serum IgM, g/L	0.8 (0.7–1.3)
C3, g/L	101.0 (87.7–111.2)
C4, g/L	22.9 (19.7–29.0)

Thirty-two patients with prognostic follow-up were divided into remission and non-remission groups according to the outcomes after 12 months of treatment. The remission group was defined as having urinary protein of <0.3 g/L and blood albumin of >40 g/L 24 h after treatment. The non-remission group was defined as not meeting one of the above conditions after treatment. The dilution ratios for serum IgG4 detection was 1:20, as previously reported [17].

### Experimental procedure

#### Antigen coupling

The CysR antigen was ultrafiltered with PBS (9600 × *g*, 10 min) for six cycles. Microspheres were subjected to oscillating ultrasound, magnetically separated and washed with 500 μL of coupling buffer, a process repeated multiple times. After the final wash, 480 μL of coupling buffer was added, followed by 10 μL of Sulfo-NHS solution (50 mg/mL). EDC solution was prepared by dissolving 100 μL of coupling buffer into the EDC tube, mixing and adding 10 μL of the resulting solution to the microspheres under light-protective conditions. After magnetic separation and further washes with 500 μL of coupling buffer, antigen were added, and the mixture was vortexed in the dark for 2 h. The microspheres were washed twice with 500 μL of wash buffer, vortexed and preserved in a microsphere preservation solution, protected from light. CysR, CTLD1 and CTLD678 antigens were conjugated to microspheres numbered 33, 7 and 52, respectively, following the same coupling protocol.

#### Biotin-conjugating antibody

The mouse anti-human IgG4 antibody was added to an Ultracel-50K ultrafiltration tube, followed by the addition of 300 μL of PBS. After centrifugation at 9600 × *g* for eight cycles, the filtrate was transferred to a new tube. The filter membrane was inverted, centrifuged at 3000 × *g*, and the filtrate was collected. An additional 100 μL of PBS was added, and the process was repeated to obtain the concentrated antibody solution. Biotin (1 mg/mL) was then added to the concentrated protein, incubated at a constant temperature for 2 h, and centrifuged. The sample was washed with 300 μL of PBS and centrifuged at 9600 × *g* for eight cycles. After further centrifugation at 3000 × *g* with the filter membrane inverted, the filtrate was collected, washed with 300 μL of PBS, centrifuged, and the purified biotin–antibody solution was stored at –20°C.

#### Selection of the appropriate anti-human IgG4 antibody concentration

Biotinylated anti-human IgG4 antibody was diluted in ratios of 1:60, 1:120, 1:240, 1:360, 1:540 and 1:720. At each dilution ratio, control group (25 μL assay buffer) and experimental group (25 μL of a high-concentration standard with appropriate dilution) were prepared for detection. The optimal dilution of the biotinylated anti-human IgG4 antibody was determined.

#### Operation procedure

First, the microsphere–antigen vortex was ultrasonicated, the perantigen-coupled microspheres were diluted to a concentration of 200 μL with the assay buffer, and 25 μL of the solution was added to each well. Then, 25 μL of buffer solution was added to the control well, and 25 μL of the standard substance or 1:20 diluted serum was added to the wells. The plate was sealed and incubated in the dark for 30 min, and the liquid was removed. Biotinylated anti-human IgG4 antibody was diluted with the assay buffer (25 μL per well), mixed, and incubated for 30 min in the dark. The board was placed on the magnetic washing machine, and the liquid was removed. SAPE was diluted to 5 μg/mL with the assay buffer (25 μL per well), the solution was mixed thoroughly, and the plate was sealed and incubated in the dark for 30 min. Finally, a Luminex 200 instrument was used in measuring the fluorescence intensity of anti-CysR/CTLD1/CTLD678–IgG4 antibodies.

### Evaluation of multiplex suspension array detection methods for anti-CysR/CTLD1/CTLD678–IgG4

#### Standard curve and sensitivity

The fluorescence intensities of different concentrations of standard solutions were measured, and standard curves for anti-CysR/CTLD1/CTLD678–IgG4 were plotted. The regression equation was obtained, and the correlation coefficient R was used to indicate the degree of correlation. The mean ($\bar X$) and standard deviation (SD) of the 0 concentration standard for 10 times was calculated. The formula for calculating the detection sensitivity of this method was detection sensitivity = Mean + 2SD.

#### Precision

Intra-assay precision: detect the fluorescence intensity and SD of low- and high-concentration samples by testing each concentration sample 10 times. Calculate the SD value as the coefficient of variation within the measurement. Inter-assay precision: test low and high concentration samples 10 times each day for 3 consecutive days, and calculate the fluorescence intensity values and SD values for each concentration sample. The SD value is the coefficient of variation between batches.

#### Recovery

The high-concentration standard substance was mixed with the known low-concentration serum (1:9), testing was repeated three times and the average value was calculated. The mean value was first calculated and then the actual concentration of the mixed solution was calculated using a standard curve. The recovery rate (%) is equal to the actual concentration/theoretical concentration multiplied by 100%.

#### Specificity

Human IgA antibody (2 mg/mL) were used in measuring the specificity of the assay. It was used as a potential disruptor, and whether cross-reactions occurred between the antibodies was determined. Each antibody was tested twice with the multiplex suspension array.

### Comparison of the multiplex suspension array and TRFIA

The serum samples of 47 patients with IMN and 20 healthy subjects were analyzed with the multiplex suspension array, and the correlation between the serum sample results and TRFIA detection results was analyzed.

### Statistical analysis

SPSS 26.0 version was used for statistical analysis, and GraphPad Prism 8.0 version was used for mapping. Schematics were drawn using Adobe Illustrator version 2023. A *P* of <.05 was considered statistically significant. Independent samples *t*-test and Kruskal–Wallis test were used to calculate the significance of the numerical differences between the groups. Spearman rank correlation coefficient was used to analyze the correlation between anti-CysR/CTLD1/CTLD678–IgG4 concentrations detected by different detection methods. Data are expressed as median (interquartile range) and mean ± SD, $\overline {\,\,X} $ ± SD.

### Ethics approval and consent to participate

This study was performed in line with the principles of the Declaration of Helsinki. The study was approved by the ethics committee of Hangzhou Hospital of Traditional Chinese Medicine (ethics approval number: 2017LH001) and was conducted in accordance with the ethical principles stated in the Declaration of Helsinki.

## RESULTS

### Selection of the appropriate anti-human IgG4 antibody concentration

**Figure 1:**
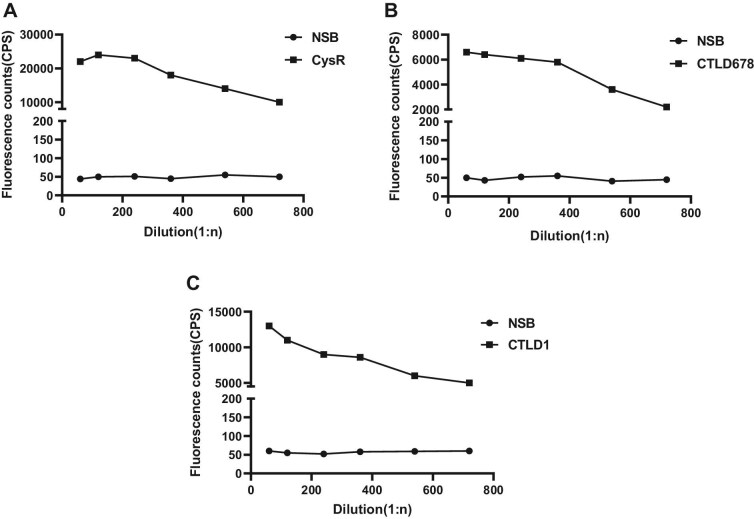
Selection of biotinylated antibody dilution ratio for (**A**) anti-CysR–IgG4, (**B**) anti-CTLD678–IgG4 and (**C**) anti-CTLD1–IgG4.

As shown in Fig. 1, after the addition of the biotinylated anti-human IgG4 antibody dilution, the fluorescence intensity of the anti-CysR/CTLD1/CTLD678–IgG4 antibody gradually decreased with increasing proportion, and IgG4 was biotinized. The highest fluorescence intensities of the three epitopes were obtained at the lowest background fluorescence intensities when the dilution ratio of the antibody was 1:120. Therefore, the dilution ratio of the biotinylated anti-human IgG4 antibody was 1:120.

### Standard curve and its sensitivity

**Figure 2:**
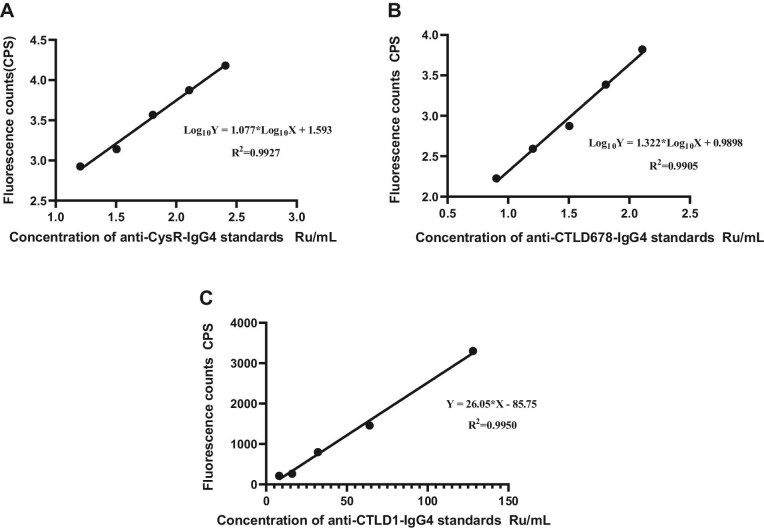
Standards curves for (**A**) anti-CysR–IgG4, (**B**) anti-CTLD678–IgG4 and (**C**) anti-CTLD1–IgG4.

The standard curve, detection range, and detection sensitivity were measured, and the results are shown in Fig. 2 and Table 2. The standard curve equations were as follows:


\begin{equation*}
\begin{array}{@{}l@{}} {\mathrm{CysR: Log}}\left( {\mathrm{Y}} \right) = {\mathrm{1}}{\mathrm{.077*Log}}\left( {\mathrm{X}} \right) + {\mathrm{1}}{\mathrm{.593}}\left( {{{\mathrm{R}}^{\mathrm{2}}} = {\mathrm{0}}{\mathrm{.9927}}} \right);\\{\mathrm{ CTLD1}} : Y = 26 {\mathrm{.05*X}} - {\mathrm{85}}{\mathrm{.75 }}\left( {{{\mathrm{R}}^{\mathrm{2}}} = {\mathrm{0}}{\mathrm{.9950}}} \right){\mathrm{;}}\\ {\mathrm{ CTLD678:Log}}\left( {\mathrm{Y}} \right) = {\mathrm{1}}{\mathrm{.322*Log}}\left( {\mathrm{X}} \right) + {\mathrm{0}}{\mathrm{.9898}}\left( {{{\mathrm{R}}^{\mathrm{2}}} = {\mathrm{0}}{\mathrm{.9905}}} \right). \end{array}\end{equation*}

**Table 2:** Sensitivity and detection range of each epitope.

	Detection sensitivity (Ru/mL)	Detection range (Ru/mL)
CysR	1.93	1.93–547.08
CTLD1	1.18	1.18–233.07
CTLD678	2.50	2.50–282.60

### Precision

The low- and high-value samples of the anti-CysR/CTLD1/CTLD678–IgG4 antibody were detected 10 times each. The results are shown in Table 3. The intra-assay coefficients of variation (CVs) of the method ranged from 4.74% to 9.48% (<10%), and the inter-assay CVs ranged from 5.13% to 13.92% (<15%), indicating good precision.

**Table 3:** Intra-assay and inter-assay precision.

		Intra-assay precision (*n* = 10)		Inter-assay precision (*n* = 10)	
	Concentration	Measured (Ru/mL)	CV (%)	Measured (Ru/mL)	CV (%)
CysR	Low	2.08 ± 0.03	9.48	2.20 ± 0.04	13.92
	High	247.85 ± 3.98	4.74	251.53 ± 4.39	5.13
CTLD1	Low	1.58 ± 0.14	7.88	1.69 ± 0.13	7.64
	High	128.86 ± 4.75	7.56	110.97 ± 6.56	10.59
CTLD678	Low	3.03 ± 0.14	8.50	3.18 ± 0.21	13.70
	High	189.68 ± 2.21	7.43	175.99 ± 2.16	7.20

### Recovery

The high-concentration standard was mixed with the known low-concentration serum in a ratio of 1:9. The concentration of the mixed solution was measured using the standard curve and was used as the actual measured concentration. The average recovery rates of the anti-CysR/CTLD1/CTLD678–IgG4 antibody were 94.89%, 93.89% and 92.72%.

### Specificity

Human IgA antibody standards were used for detection and as potential interferer during the measurement of detection method's specificity. Whether cross-reaction between antibodies occurred was determined. The results showed that the fluorescence intensity was far lower than the sensitivity of the detection method, indicating that no cross-reaction occurred in the whole system. The results showed that the antigens and antibodies had high specificity.

### Comparison of multiplex suspension array and TRFIA

**Figure 3:**
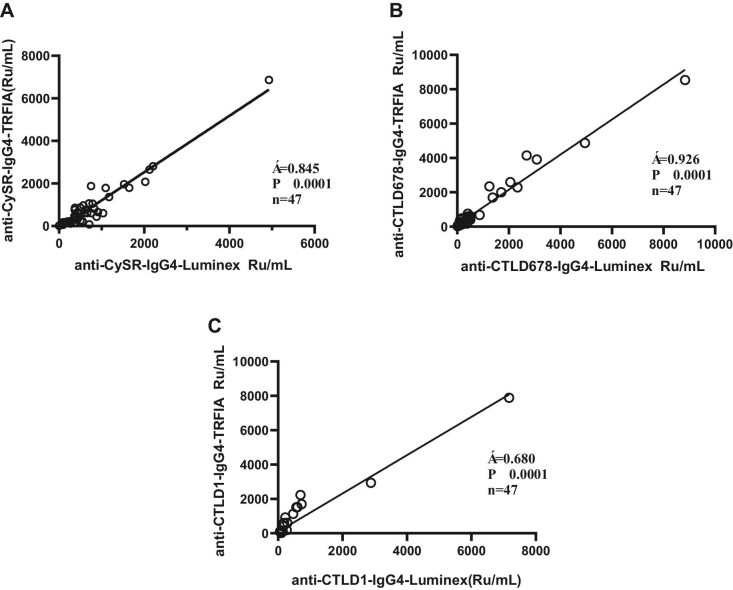
Comparison of different methods for detection concentrations of (**A**) anti-CysR–IgG4, (**B**) anti-CTLD678–IgG4 and (**C**) anti-CTLD1–IgG4.

Serum samples from 47 patients and 20 healthy controls were analyzed. The correlation between the results of multiplex suspension array and TRFIA was analyzed. As shown in Fig. 3, the related equations of the two detection methods are as follows:


\begin{eqnarray*}
&&{\mathrm{CysR: Y = 1}}{\mathrm{.324*X}} - {\mathrm{120}}{\mathrm{.0; CTLD1: Y = 1}}{\mathrm{.114*X + 88.72;}}\\ &&{\mathrm{ CTLD678:Y = 1}}{\mathrm{.022*X + 104}}{\mathrm{.8}}{\mathrm{.}}
\end{eqnarray*}

### Clinical application

**Figure 4:**
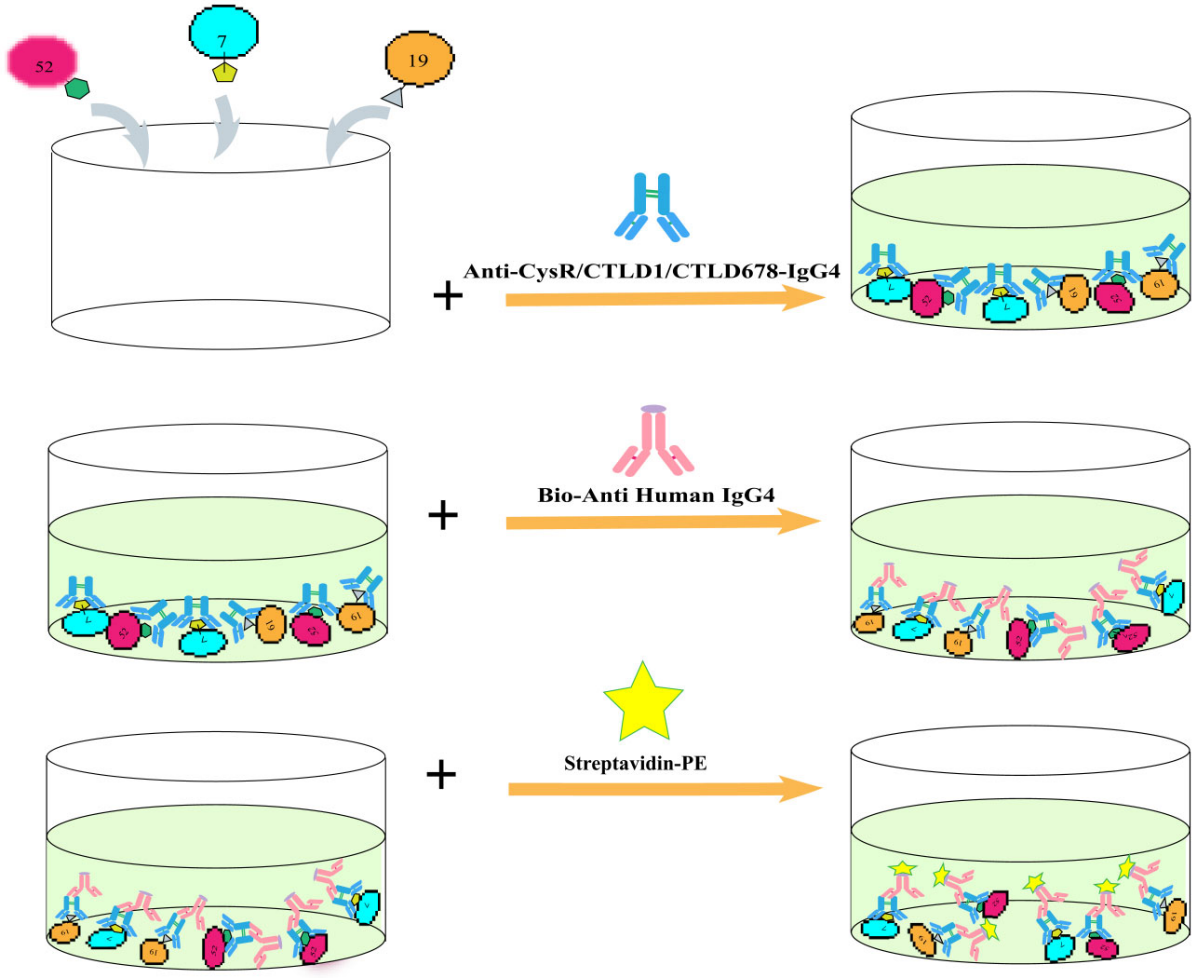
Schematic of anti-CysR/CTLD1/CTLD678–IgG4 multiplex suspension array.

A method for the simultaneous and specific detection of anti-CysR/CTLD1/CTLD678–IgG4 was set (Fig. 4). This method was used in determining the serum levels of PLA2R antibody–positive in patients with IMN and healthy individuals. Serum anti-CysR/CTLD1/CTLD678–IgG4 levels in patients with IMN were significantly higher than those in healthy subjects. The cut-off value is calculated as $\bar X$ + 2SD ($\bar X$ that is, the mean concentration of anti-CysR/CTLD1/CTLD678–IgG4 in the serum of healthy subjects; SD: the standard deviation of the concentration of anti-CysR/CTLD1/CTLD678–IgG4 in the serum of healthy subjects). Concentrations above the threshold were considered positive. In this study, we determined that the cut-off values of anti-CysR–IgG4, anti-CTLD1–IgG4 and anti-CTLD678–IgG4 were 6.30, 12.38 and 10.06 Ru/mL, respectively. Among the 32 PLA2R-positive patients, the positive detection rates of the CysR epitopes, CTLD1 epitopes, and CTLD678 were 100%, 34% and 75%, respectively.

### CysR/CTLD1/CTLD678 epitope spreading correlates with disease severity

Patients were divided into two groups based on whether they achieved clinical remission at the 12-month follow-up. A total of 20 patients achieved remission, while 12 did not. The concentration of the anti-CysR/CTLD1/CTLD678–IgG4 level were significantly higher in the non-remission group compared with the remission group. A cut-off value was determined by the Youden index of Table 4. The threshold values of anti-CysR/CTLD1/CTLD678–IgG4 were 361.9 Ru/mL, 42.76 Ru/mL and 64.40 Ru/mL, respectively. The cut-off values were set between the remission group and the non-remission group, only the cut-off value of CTLD1 was significantly different (*P *= .0001), and CTLD1 concentration >42.76 Ru/mL is considered to be associated with poor prognosis.

**Tabel 4:** The threshold between the response group and the non-response group.

		Cut-off	Sensitivity	Specificity	AUC	95% CI	*P*-value
Remission vs no-remission	CysR (Ru/mL)	361.9	0.8571	0.5000	0.6500	0.4614 to 0.8386	.1416
	CTLD1 (Ru/mL)	42.76	1.000	0.9500	0.9500	0.8545 to 1.000	<.0001
	CTLD678 (Ru/mL)	64.40	0.8571	0.2500	0.5286	0.3297 to 0.7274	.7795

CI, confidence interval; AUC, area under the curve.

Moreover, epitope grouping was as follows: all the 32 positive patients (100.00%) responded to CysR fragments (Table 5). Therefore, when no other epitope group existed, as in the case of the CysR fragment response, we grouped the above queues. The groups were as follows: Group 1, which responded to the CysR fragment only; Group 2, which responded to CTLD678; and Group 3, which responded to CTLD678 and CTLD1 simultaneously. In Group 1, all the patients entered remission (Table 6). In Groups 2 and 3, only 10 (72%) and 3 (27%) patients had remission, respectively. Group 3 constituted 73% of the eight patients who were not in remission, indicating that the clinical indication was apparent when the epitope migrated to CTLD1; therefore, CTLD1 may respond to epitope migration and increase the severity of IMN.

**Tabel 5:** Epitope grouping of all enrolled patients in this study (*n* = 32).

	Epitopes recognized	Total (*n* = 32)
	CysR	7/32
One epitope respond	CTLD1	
	CTLD678	
	CysR + CTLD1	
Two epitopes respond	CysR + CTLD678	14/32
	CTLD1 + CTLD678	
Three epitopes respond	CysR + CTLD1 + CTLD678	11/32

**Tabel 6:** Relationship between spreading and PLA2R domain groups.

	Remission (*n* = 20)	Non remission (*n* = 12)	*P*-value
Sex	11 M/9 F	8 M/4 F	ns
Age	51 ± 14	54 ± 14	.420
24-h urinary protein (g/day)	4.5 ± 3.5	6.3 ± 2.8	.152
Serum albumin (g/L)	25.2 ± 5.6	23.4 ± 4.2	.355
Serum creatinine (µmol/L)	67.8 ± 17.8	102.0 ± 65.0	.034
eGFR, mL/min/1.73 m^2^	94.09 ± 17.2	75.1 ± 35.0	.048
CysR (*n* = 7)	7 (100%)		ns
CysR+CTLD678 (*n* = 14)	10 (72%)	4 (28%)	ns
CysR + CTLD1 + CTLD678 (*n* = 11)	3 (27%)	8 (73%)	ns

Normal values are expressed as mean ± SD, and abnormal values are expressed as quartiles. In this study, the clinical data of patients were divided into remission stage and non-remission stage, 10 patients were in remission stage and 7 patients were not in remission. The 24-h proteinuria and albumin were significantly higher in the non-remission group at the time of diagnosis.

M, male; F, female; ns, not significant; eGFR, estimated glomerular filtration rate.

## DISCUSSION

In the progression of IMN, CysR is the first epitope recognized on PLA2R protein molecules, followed by non-cross-reactive epitopes CTLD1 and CTLD7 or by dominant epitopes on neighboring molecules through intermolecular diffusion [8]. Patients with autoantibodies that recognize only the CysR domain showed better prognoses than patients with epitope spreading. Conversely, patients identified with domains other than CysR were thought to have poor prognoses. However, researchers have different views about the role of PLA2R epitope spreading in patients with PLA2R-associated IMN. Thus, whether the PLA2R domain epitope characteristics and anti-CTLD1 and CTLD678 antibody series levels can be used as prognostic indicators requires further study.

The main methods for detecting different epitopes in serum samples of patients with IMN are western blot, ELISA and TRFIA [8, 15, 16, 18]. Western blot is qualitative or semiquantitative analysis, and the operation is complicated. ELISA is a semiquantitative method usually performed under the condition of non-denaturation. Although recent studies have shown that TRFIA enables quantitative analysis with superior sensitivity and a broader detection range compared with earlier ELISA methods, TRFIA is limited to the separate detection of individual antibodies. This inability to maintain a consistent serum environment can lead to significant measurement errors. To address this issue, the present study developed a multiplex suspension array method for simultaneous detecting anti-CysR/CTLD1 and CTLD678–IgG4 antibodies. This approach allows serum samples to remain under consistent reaction conditions while simultaneously quantifying specific IgG4 antibodies targeting three structural domains of PLA2R in patients with IMN. The method effectively enhances detection efficiency and reduces measurement errors.

We simultaneously and quantitatively measured anti-CysR/CTLD1/CTLD678–IgG4 levels using a multiplex suspension array. This method maintains a stable liquid environment conducive to the proper conformation of macromolecules and minimizes testing error by analyzing at least 2500 microspheres per indicator and using the median of 100 randomly selected readings. The antigens of the three PLA2R epitopes were coupled to magnetic beads, forming fluorescent immune complexes through sequential addition of serum, biotinylated anti-human IgG4 antibody and streptavidin–PE complexes. The microspheres were classified by fluorescence signals, and bound analytes were quantified based on PE emission values. This approach offers short incubation times, low sample requirements and high throughput.

We analyzed 32 PLA2R antibody–positive patients with IMN, who had clinical information. The IgG4 anti-PLA2R subclass in patients were analyzed because IgG4 is the major subclass of the PLA2R antibody and is associated with disease activity [8, 13, 19, 20]. Among these patients, the positive detection rates of the CysR epitopes, CTLD1 epitopes, and CTLD678 were 100.00%, 35.30% and 88.23%, respectively. The findings demonstrate that the positive rate of the CysR epitope to the CTLD1 epitope decreased gradually, indicating that epitope spreading occurred.

In addition, all the patients in Group 1 experienced remission. In Group 2, all 14 patients responded to the CysR and CTLD678 epitopes: 10 achieved clinical remission and 4 were not in remission. The remission rate in Group 3 (responded to the CTLD1 domain) was 27%. This presented that CTLD1 may appear last in the course of IMN, and the sequence of epitope migration may be from CysR to CTLD678 and finally to CTLD1. When the epitope migrated from CysR to CTLD678, the probability of spontaneous remission decreased in patients. When the epitope was extended to CTLD678 and CTLD1, the urinary protein content in the patients increased, and the clinical indication of patients increased in severity. The results showed that the PLA2R epitope migration is associated with remission. In this study, only CysR and CTLD678 responses were obtained, and the clinical symptoms of patients were relatively mild. Their conditions may have been more likely to be alleviated after treatment. When CTLD1 epitopes respond simultaneously, patients are more likely to experience difficulty in entering remission during treatment. Therefore, anti-CTLD1–IgG4 level may be an important indicator for determining whether patients with IMN are in remission. Our study found that the CTLD1 epitope is associated with the progression of membranous nephropathy, and the levels of CTLD1–IgG4 antibodies may influence the remission status of patients. The CTLD1 response rate was 67% in the non-remission group, prompting us to hypothesize that CTLD1 may serve as a biomarker for disease severity and a predictor of clinical response. Furthermore, we identified a cut-off value of 42.76 Ru/mL for the CTLD1 domain to distinguish between the remission and non-remission groups. Patients with CTLD1 levels exceeding this threshold are suggested to have a poorer prognosis and a reduced likelihood of achieving remission. These findings are consistent with the conclusions of a recent retrospective study conducted by Liu's team, which demonstrated that PLA2R–CTLD1–IgG4 levels had superior predictive value for proteinuria remission at 6 months compared with total anti-PLA2R–IgG antibody levels [21]. Interestingly, this observation deviates from the previously established notion that epitope spreading follows a linear pattern [8].

**Figure 5:**
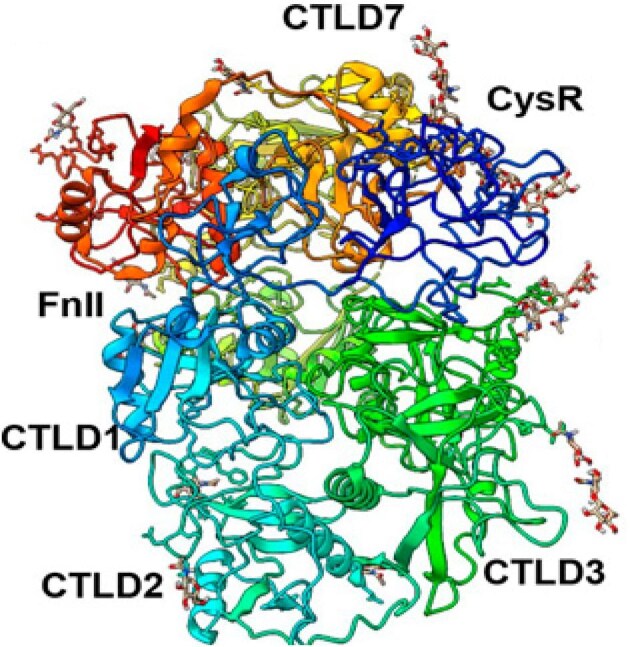
High-resolution cryoEM structure of human PLA2R reveals a different overall domains arrangement. [The original picture is provided by Maryline Fresquet, published in [22], licensed under CC by 4.0.]

**Figure 6:**
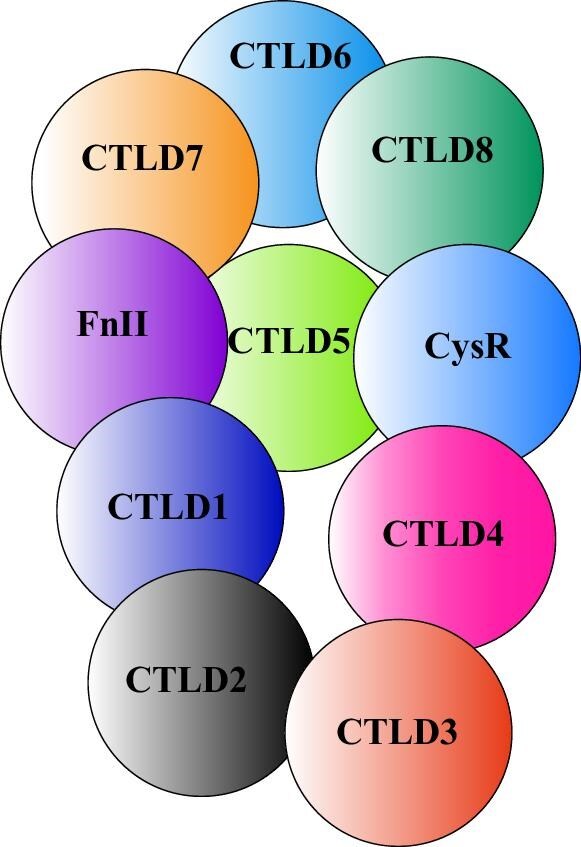
Corresponding schematic model of PLA2R showing the colored domains and their arrangement. Image sourced from reference [22].

This result may be attributed to PLA2R molecular epitopes including not only linear peptides but also their stereospatial conformation. According to Fresquet *et al*. [12] the epitopes within the PLA2R molecule are highly conformational, and their exposure depends on the protein's three-dimensional structure and interdomain interactions. The epitope CTLD1 may located near the central structure of PLA2R and participates in key conformational epitopes, which may make it important in the immune response. The positions of PLA2R conformational epitopes are shown in Figs 5 and 6. As the PLA2R epitope exposure depends on the three-dimensional spatial structure, the spatial distance between CysR and CTLD1 is larger than that between CysR and CTLD678. Thus, the order of epitope migration during disease progression may not follow a simple linear rule. Moreover, the study by Liu *et al*. [21] demonstrated that elevated CTLD1–IgG4 levels were independent predictors of lower remission rates at 6 months. These antibodies showed stronger predictive value for proteinuria remission than total anti-PLA2R–IgG levels, highlighting the role of CTLD1 in disease progression. This aligns with the notion that advanced epitope spreading, especially to CTLD1, marks a more severe immunological state, correlating with poorer remission outcomes in patients.

In summary, this study established a multiplex suspension array–based technology that can simultaneously measure the levels of anti-CysR/CTLD1/CTLD678–IgG4 in small volumes (5 μL) of serum samples. It has the advantages of having consistent inspection environment, short detection time and a small sample volume. Furthermore, our findings suggest that the epitope spreading of PLA2R molecules likely follows a progression starting from CysR to CTLD678 and eventually to CTLD1, rather than proceeding in a simple linear sequence.

This study has several limitations that should be acknowledged. First, the relatively small sample size and single-center design may limit the generalizability of our findings to larger, more diverse populations or different ethnic groups. Second, while the multiplex suspension array technology demonstrated high sensitivity and specificity, its relatively high equipment costs, lower adoption rate compared with established methods such as TRFIA and ELISA, and the lack of full automation. Future studies with larger, multicenter cohorts and advancements in automation could help address these limitations and further validate our findings.

## Data Availability

The datasets used and/or analyzed in the current study are available from the corresponding author upon reasonable request.
